# What accounts for the rise of low self-rated health during the recent economic crisis in Europe?

**DOI:** 10.1186/s12939-019-0926-1

**Published:** 2019-01-28

**Authors:** Michal Brzezinski

**Affiliations:** Faculty of Economic Sciences, University of Warsaw, Dluga 44/50, 00-241 Warsaw, Poland

**Keywords:** Self-rated health, Economic crisis, Decomposition, Oaxaca-Blinder, Unemployment, Material deprivation

## Abstract

**Background:**

A growing literature investigates health effects of the recent economic crisis. This study examines how different economic mechanisms affected low self-rated health (SRH) in Europe over the crisis period (2008–2011). We measure changes in low SRH over 2008–2011 and analyze how they are accounted for by changes in household income levels and income distribution (income poverty, income inequality), labour market developments (increasing unemployment, falling employment, changes in labour market inactivity), and non-income poverty (material deprivation).

**Methods:**

We use balanced panel data for 2008–2011 covering 26 European countries and 43,456 participants. The data come from longitudinal 2011 European Union Statistics on Income and Living Conditions (EU-SILC) database. Increases in low SRH incidence over time are decomposed into the contributions of changes in the distribution of covariates and changes in returns to the covariates. Main covariates include household income and its distribution, labour market developments, and non-income poverty (material deprivation). The decompositions are performed using a detailed non-linear multivariate regression-based decomposition methodology.

**Results:**

Low SRH incidence increased in Europe during the crisis by almost 2 percentage points, and by 3.7 percentage points in case of the Baltic countries. Decomposition analysis shows that: 1) decreasing household incomes and changing income distribution had no impact on low SRH incidence, 2) rise of material deprivation accounts for a significant portion (12%) of the overall growth in low SRH rates (27% for the Baltic countries), 3) decreasing levels of full-time and part-time employment as well as transitions to unemployment, economic inactivity, disability, or retirement account jointly for about 21% of the rise in low SRH in Europe (73% for Baltic countries).

**Conclusion:**

Together, the recession-related economic factors account for about 33% of the increase in low SRH incidence in Europe during the crisis, and for about 100% of the increase in the Baltic countries. Public health policy during recessions should focus also on reducing material deprivation through free or subsidized access to public services, public housing, and other means.

**Electronic supplementary material:**

The online version of this article (10.1186/s12939-019-0926-1) contains supplementary material, which is available to authorized users.

## Introduction

The recent economic crisis that started in 2008, often called the Great Recession (GR), was the worst global economic recession since the Great Depression of the 1930s. For many European countries, reduced economic activity has brought decreasing household incomes, increased unemployment or labor market inactivity, and growing material deprivation. Most of the countries experienced loses in average household incomes over 2008–2013 with Iceland (28% decline) and Greece (40%) being the biggest losers. Unemployment rates grew over 2008–2013 in all European countries except Germany. Income poverty anchored at 2008 poverty line increased over 2008–2013 in almost half of European countries and more than doubled for Greece and Iceland. Severe material deprivation rate, which captures the proportion of people whose living conditions are severely affected by lack of resources also grew in most of the European countries, and it more than doubled for Iceland, Luxembourg and Malta. Several governments responded to the crisis by implementing policies of austerity or fiscal consolidation, which could have further amplified negative impact of the crisis on health outcomes. For example, governments in Greece, Ireland, Latvia and Portugal have made large cuts to public health spending [1].

Theoretical considerations suggest several pathways leading from worsening economic conditions to health deterioration [2, 3]. Lower employment and reduced control over economic resources due to a recession can increase psychological stress and risk taking with negative impact on both physical and mental health. On the other hand, lower earnings and reduced economic activity may have health-improving effects through lower alcohol consumption, reduced obesity and smoking, and increased physical exercise.

Several previous empirical studies have investigated the impact of the GR on various health outcomes such as suicide rates [4], fertility [5], mortality [6, 7] or depression [8–10]. Some papers found evidence for the negative impact [3, 11, 12], while others reported rather small or insignificant effects [6–8]. Many studies focused on changes in self-rated health (SRH) in individual European countries such as Greece [9, 13, 14], Spain [15–17], the UK [18], or Estonia, Lithuania and Finland [19]. Tøge and Blekesaune [20] used longitudinal data from the European Union Statistics on Income, Social Inclusion and Living Conditions (EU-SILC) data to show that becoming unemployed during the GR had a negative effect on SRH. However, this effect diminishes in the first few years after becoming unemployed. Abebe et al. [3] found that incidence of fair and poor SRH has increased during the GR in a sample of working-age population living in 23 European countries. They also found that the micro- and macro-level covariates of low a and fair SRH did not change significantly between the pre-recession period (2005–2007) and the recession period (2008–2011).

The existing empirical literature has so far analysed the impact of the GR on health mostly through labour market developments including growing unemployment rates. However, health during economic crises can be also affected through other channels such as declining household incomes and growing income and non-income poverty (material deprivation). Research has shown that income and its distribution as well as material deprivation can have a significant effect on health [21–24]. The present paper fills the gap by estimating how the GR affected low SRH rates in 26 European countries through three different economic mechanisms: changes in household income levels and income distribution (income poverty, income inequality), labour market developments (increasing unemployment, falling employment, changes in labour market inactivity), and non-income poverty (material deprivation). The analysis is conducted using a balanced panel data set for 2008–2011 coming from the EU-SILC survey. The contribution of the three studied economic mechanisms to the rise of low SRH rates is estimated using a regression-based decomposition methodology [25–28]. The paper does not aim at measuring the causal impact of the studied factors on low SRH, but rather attempts to discover whether growing incidence of low SHR in Europe during the GR can be accounted for not only by transitions of individuals from employment to unemployment, but also by transitions to income and non-income poverty. This approach allows to measure the relative strength of the association between different economic recession-related factors and low SRH in Europe. The results may be useful for a policymaker concerned with designing policies mitigating health impacts of recessions in a most efficient way.

## Methods

### Decomposition technique

To identify factors that are associated with changes in low SRH over time, we use a variant of multivariate regression-based Oaxaca-Blinder decomposition technique [25, 26], which is routinely applied to account for changes in mean socio-economic outcomes (e.g. wages, health statuses) between various socio-economic groups (e.g. men versus women) or changes in these outcomes over time. The methodology allows to partition the observed difference in a given outcome into a component attributable to differences in the observed *characteristics* (i.e. individual demographic, economic or educational attributes) and a component attributable to differences in the *returns* to these characteristics. The former component is often called an “explained” part of the difference in the outcome, while the latter component is called an “unexplained” one. The Oaxaca-Blinder methodology has been applied to analyse various issues in the health literature [27, 29, 30]. In our context, we are interested in explaining changes in the probability of reporting low level of SRH, Pr(SRH ≤ *k*), where *k* is the chosen cut-off level separating individuals with low SRH from individuals with medium or high level of SRH. In our empirical analysis below, we define *k* as “fair” SRH status. Under a logit probability model, the likelihood of a person *i* having a low level of SRH in time *t*, ${P}_i^t(k)$, can be written as:

1 $$ {P}_i^t(k)=\Pr \left({\mathrm{SRH}}_i^t\le k\right)=F\left({X}_i^t{\widehat{\beta}}^t\right)=\frac{\exp \left({X}_i^t{\widehat{\beta}}^t\right)}{1+\exp \left({X}_i^t{\widehat{\beta}}^t\right)} $$

where *F* is the logistic probabilistic cumulative distribution, ${X}_i^t$ is a vector of independent variables or characteristics describing the individual *i* in period *t*, and ${\widehat{\beta}}^t$ is the vector of estimated coefficients or returns to these characteristics. In our empirical analysis, the logit regressions (1) are estimated separately for 2008 and 2011. The vector *X* includes our choices for variables related to the recession, control variables, and country dummies (see next section for details).

For the logit model, the low SRH rate in period *t*, *H*^*t*^, is equal to the average predicted probability of having low SRH:

2 $$ {H}^t=\overline{F\left({X}_i^t{\widehat{\beta}}^t\right)}, $$

where *F* is the logistic probabilistic cumulative distribution, ${X}_i^t$ is a vector of independent variables or characteristics describing the individual *i* in period *t*, and ${\widehat{\beta}}^t$ is the vector of estimated coefficients or returns to these characteristics in period *t*. Using property (2), the difference between the low SRH rates observed in two periods *t* = 0 and *t* = 1,

3 $$ {H}^1-{H}^0=\overline{F\left({X}^1{\beta}^1\right)}-\overline{F\left({X}^0{\beta}^0\right),} $$

can be decomposed into the aggregate characteristics or “explained” effect, *E*, and aggregate coefficients or “unexplained” effect, *C*:

4 $$ {H}^1-{H}^0=E+C=\left\{\overline{F\left({X}^1{\beta}^1\right)}-\overline{F\left({X}^0{\beta}^1\right)}\right\}+\left\{\overline{F\left({X}^0{\beta}^1\right)}-\overline{F\left({X}^0{\beta}^0\right)}\right\}. $$

The aggregate “explained” and “unexplained” effects can be further decomposed into individual effects *E*_*g*_ and *C*_*g*_, *g* = 1, …, *G*, representing the contributions of an individual *g*th element of *X* to, respectively, the aggregate effects *E* and *C*:

5 $$ {H}^1-{H}^0=E+C={\sum}_{g=1}^G{W}_{\Delta  X}^gE+{\sum}_{g=1}^G{W}_{\Delta  \beta}^gC, $$

where *W* are the appropriate weights obtained by approximating the value of the average of function $\overline{F\Big( X\beta}\Big)$ with the value of the function evaluated at the average values of the elements of *X*, $F\left(\overline{X}\beta \right)$, and applying a first-order Taylor linearization of equation (4) around ${\overline{X}}^1{\beta}^1$ and ${\overline{X}}^0{\beta}^0$ [28, 31, 32]. This approach is easy to interpret, straightforward to compute, invariant to the order of inclusion of variables into the decomposition, as well as invariant to the choice of the reference (or omitted) category, when categorical variables are used as elements of *X.*

### Data on SRH and covariates

We use data on SRH and its correlates from the longitudinal 2011 European Union Statistics on Income and Living Conditions (EU-SILC) database. These data refer to the period 2008–2011, which covers most of the crisis period in Europe. We use a balanced four-year panel sample of individuals that were interviewed in each year between 2008 and 2011. The sample covers 26 EU countries; no longitudinal information for Germany and Ireland was available for the 2008–2011 period. In all analyses, we apply EU-SILC individual longitudinal weight to account for survey non-response and longitudinal attrition. Standard errors for all estimates are clustered at the country level.

The wording of the SRH question in EU-SILC is as follows: “How is your health in general?”. The answers are ranked on a 5-point scale: 5 = “very good”, 4 = “good”, 3 = “fair”, 2 = “bad”, and 1 = “very bad”. We define low SRH as reporting “fair” or worse health. Our analysis is performed for individuals between 18 and 64 years of age. We excluded proxy interviewees (respondents who had someone else answer the questions for them). Table 1 shows descriptive statistics for all variables used.

**Table 1:** Descriptive statistics for the pooled sample of 26 EU countries

	2008		2011	
	Mean	SD^a^	Mean	SD^a^
SRH status: 1–5 scale, 1 = very bad, 5 = very good	3.908	0.852	3.844	0.878
Very bad SRH	0.009	0.094	0.013	0.112
Very bad or bad SRH	0.060	0.238	0.076	0.265
Very bad, bad or fair SRH	0.267	0.442	0.285	0.452
Female	0.591	0.492	0.563	0.496
Age	44.343	12.043	46.004	12.412
Real equivalent household income (Euro in 2004 prices)	18,824.3	14,381.9	16,945.3	14,837.6
Poor (income < 60% of the median)	0.154	0.361	0.173	0.378
Rich (income > 200% of the median)	0.083	0.276	0.083	0.276
Relatively poor	0.116	0.320	0.125	0.330
Relatively rich	0.057	0.231	0.059	0.236
Material deprivation	0.154	0.361	0.178	0.382
Primary education and less	0.114	0.317	0.102	0.303
Lower secondary education	0.154	0.361	0.155	0.362
Upper secondary education	0.462	0.499	0.472	0.499
Tertiary education	0.270	0.444	0.271	0.444
Married	0.587	0.492	0.610	0.488
No longer married	0.135	0.341	0.138	0.344
Never married	0.279	0.448	0.252	0.434
Employed, full-time	0.544	0.498	0.519	0.500
Employed, part-time	0.125	0.331	0.107	0.310
Unemployed	0.058	0.233	0.072	0.259
Retired	0.126	0.332	0.146	0.353
Disabled	0.041	0.198	0.040	0.196
Inactive	0.106	0.308	0.116	0.320
Densely populated area	0.452	0.498	0.468	0.499
Intermediate populated area	0.319	0.466	0.289	0.453
Thinly populated area	0.257	0.437	0.316	0.465
Observations	43,456			

a: Standard deviation

In our decomposition analyses aiming at identification of factors that are associated with changes in low SRH, we use several variables that can affect SRH. The standard controls include age, sex, educational attainment, marital status, degree of urbanization, and country dummies. We expect that the impact of the economic crisis on low SRH can be captured using three types of economic variables: income and its distribution [21, 33], labour market status [20], and material deprivation status [34, 35]. We use real equivalized disposable (after tax and transfer) income expressed in 2004 prices as our main income variable. The impact of income poverty and inequality [36–38] is accounted for by binary indicators of income poverty (income less than 60% of the median income in the country) or richness (income higher than 200% of the country’s median income). In order to verify the relative income hypothesis, which posits that individual’s health is affected rather by her relative than absolute income [39, 40], we use binary indicators of being relatively poor (rich) defined as having income lower (higher) than 60% (200%) of her reference income within a country (average income of individuals with the same gender, age class and education category). Material deprivation is measured by binary indicator variable taking the value of one if a person cannot afford at least three of the following nine items: mortgage, rent or utility payments, one week’s holiday away from home, a meal with meat every second day, unexpected financial expenses, a telephone (including mobile telephone), a colour TV, a washing machine, a car and heating to keep the home sufficiently warm. The correlation between absolute income poverty (richness) and relative poverty (richness) measures, as well as between poverty and material deprivation indicators is moderate (ranging from 0.2 to 0.6). Therefore, we can include all three sets of variables related to poverty in the model specification without causing multicollinearity (the average variance inflation factor across all predictors is 1.5). Finally, the role of rapid and profound labour market perturbations during the GR is accounted for by dummy variables describing whether a person was a full-time or part-time employee, unemployed, retired, disabled or inactive due to other reason.

## Results

Table 2 presents estimates for low SRH rates and their changes between 2008 and 2011, calculated separately for the sample of all EU countries, severe recession countries and the Baltic countries, which were hit particularly hard by the GR. In case of all EU countries, we observe that the low SRH incidence grew over 2008–2011 by almost 2 percentage points. The incidence of low SRH increased even more for the Baltic countries (3.8 percentage points). Estonia and Lithuania have experienced increases in low SRH rates by more than 4 percentage points. On the other hand, we observe a small decline, although statistically insignificant, in low SRH incidence in the group of severe recession countries. It is likely that the negative impact of recession on health in the Baltics was amplified by cuts in healthcare funding and reductions in coverage of health services [19]. These results are consistent with Abebe et al. [3] who have found that during the crisis low SRH incidence was either increasing or stable in most of the EU countries.

**Table 2:** Estimates of low SRH^a^ rates for various samples of European countries, 2008–2011

Sample	Low SRH (%) in 2008	Low SRH (%) in 2011	Change in low SRH	% change in low SRH	*P*-value^b^
All EU countries	26.66 (0.2)	28.52 (0.2)	1.9	7.0	0.000
Severe recession countries^c^	24.44 (0.4)	23.89 (0.4)	−0.6	−2.3	0.315
Baltic countries	46.50 (0.8)	50.29 (0.8)	3.8	8.2	0.001

Standard errors clustered by country appear in parentheses

a: Low SRH is defined as the proportion of population with very bad, bad or fair SRH

b: *p*-value for the test of equality between the estimates of low SRH rates for 2008 and 2011

c: Bulgaria, Cyprus, Denmark, Estonia, Greece, Iceland, Latvia, Lithuania, Slovenia, Spain

Decompositions of increases in low SRH rates over 2008–2011 for all EU countries and the Baltic countries are presented in Table 3. In both cases, the “explained” part of the decomposition is highly statistically significant. It accounts for as much as 1.8 percentage point increase in the low SRH rate in the sample of all EU countries (93% of the overall rise in low SRH incidence over 2008–2011). For the Baltic countries, the contribution of the “explained” part to the increase in low SRH rate is actually higher than the overall rise in low SRH incidence. This happens as the “unexplained” part due to the changing returns to covariates is negative (it reduces the incidence of low SRH). However, the “unexplained” component is statistically insignificant. Overall, these results suggest that our decomposition approach does a good job in accounting for the increases in low SRH incidence in Europe during the GR.

**Table 3:** Decomposition of changes in low SRH^a^ rates for various samples of European countries, 2008–2011

	All EU countries		Baltic countries	
	Estimate	% of change in low SRH	Estimate	% of change in low SRH
Change in low SRH	1.935^***^ (0.514)	100	3.790^***^ (1.229)	100
Total “explained” effect (E)	1.802^***^ (0.291)	93.1	4.771^***^ (0.460)	125.9
Total “unexplained” effect (C)	0.133 (0.616)	6.9	−0.981 (1.325)	−25.9
*Individual characteristics effects*				
Real equivalent income	0.205 (0.180)	10.6	0.372 (0.342)	9.8
Poor	0.002 (0.023)	0.1	0.001 (0.073)	0.0
Rich	−0.000 (0.000)	− 0.0	0.002 (0.002)	0.0
Relatively poor	0.005 (0.010)	0.2	0.018 (0.043)	0.5
Relatively rich	0.000 (0.003)	0.0	−0.065 (0.048)	−1.7
Material deprivation	0.224^***^ (0.019)	11.6	1.011^***^ (0.186)	26.7
Employed full time	0.345^***^ (0.036)	17.8	1.332^***^ (0.115)	35.2
Employed part time	0.168^***^ (0.028)	8.7	−0.184^***^ (0.046)	−4.8
Unemployed	−0.104^***^ (0.024)	−5.4	−0.734^***^ (0.114)	−19.4
Retired	0.091^***^ (0.031)	4.7	0.780^***^ (0.162)	20.6
Disabled	−0.028*** (0.002)	−1.5	0.896^***^ (0.207)	18.0
Inactive	−0.059^***^ (0.015)	−3.0	0.896^***^ (0.207)	23.6
Other factors^b^	0.955^***^ (0.322)	49.3	0.661^***^ (0.193)	17.4
Observations	43,456		3719	

Standard errors clustered by country appear in parentheses; ^*^p < 0.1, ^**^p < 0.05, ^***^p < 0.01

a: Low SRH is defined as the proportion of population with very bad, bad or fair SRH

b: Other factors include sex, age, education, marital status, degree of urbanization and country dummies

Table 3 presents also individual characteristics effects, which identify contributions of changes in the distribution of SRH covariates to the overall increases in low SRH. Surprisingly, we do not find any role for changes in income level or its distribution as captured by indicators of being absolutely (or relatively) poor or rich. On the other hand, increasing material deprivation contributed significantly to the rise in low SRH rates. In case of all EU countries, the characteristics effect associated with the growth of material deprivation (by 2.4 percentage points over 2008–2011, see Table 1) accounts for about 0.2 percentage point rise in the low SRH rate (or 12% of the overall increase in low SRH incidence). For the Baltic countries, the size of the effect is larger and reaches 27% of the total rise of the low SRH rate.

All individual characteristics effects related to labour market status are highly significant. Full-time and part-time employment rates in the EU have jointly fallen over 2008–2011 by 4.3 percentage points. These labour market perturbations contributed to the rise in low SRH rate by about 0.5 percentage point (27% of the increase in the overall low SRH rate). For the Baltic countries, declines in employment rates account for 1.1 percentage point increase in the low SRH incidence. The results suggest also that increasing rates of unemployment during the crisis had a small (0.1 percentage point), statistically significant effect on reducing low SHR rates in the EU (and somewhat larger effect in case of the Baltic countries). This counterintuitive result is due to weakening of the association between unemployment and the probability of reporting low SRH during the recession (see full regression results in the Additional file 1: Table A1). While in 2008 unemployment was a relatively strong statistically significant positive predictor of low SRH, it has become an insignificant correlate in 2011. Transitions to retirement during recession were increasing low SRH rates for both samples. Effects associated with disability and economic inactivity played a smaller role in case of all EU countries but were more sizable for the Baltics.

## Discussion

The main findings from the decomposition analysis are as follows. First, we do not find any impact of decreasing household incomes and changing income distribution on growing incidence of low SRH in Europe. A possible explanation for this result is that the impact of (absolute and relative) incomes and income inequality on health is empirically small or even negligible [21, 33, 40, 41]. It is also worth recalling here that in the short-run the impact of the GR on income inequality and poverty rates in Europe was rather small [42]. Second, we find that the rise of non-income poverty (material deprivation) accounts for a significant portion (12%) of the overall growth in low SRH in Europe during recession. The effect is even larger (27%) for of the Baltic countries. Third, decreasing levels of full-time and part-time employment as well as transitions to unemployment, economic inactivity, disability, or retirement account jointly for about 21% of the rise in low SRH in Europe, and for about 73% in case of the Baltic countries. Together, the recession-related economic factors account for about 33% of the increase in low SRH incidence in Europe, and for about 100% of the increase in the Baltic countries. While some previous studies have investigated the relationship between transitions from employment to unemployment and SRH during the GR [3, 20], the present paper explores additionally the role of transitions from employment to retirement, disability, or inactivity. The overall effect associated with these transitions account for as much as 62% of the rise of low SRH in the Baltic countries (see Table 3). Future studies should devote more attention to the problem of how recessions affect health of those who are retired, disabled or economically inactive.

An important novel insight of the present paper is that growing material deprivation can be an important factor worsening health during recessions. This calls for both more research on the relationship between material deprivation (or other measures of non-income poverty) and health, as well as for reconsideration of policies directed at reducing material deprivation. Such policies could include progressive taxation, free or subsidized access to public services, active labour market interventions, public housing, and others [23].

This study has a number of limitations. First, it relies on self-reported health data, which are subjective and can have limited comparability across time and various groups in the society [43]. Second, since the panel dimension of the EU-SILC data is rather short (4-years), it does not allow for studying health effects of the GR over the longer period. Third, although we were able to control for many socio-economic characteristics of individuals, there are some omitted factors such as subjective economic stress, health behaviours or psychological justification mechanisms [20] that could be mediators between the economic effects of the recession and reported health statuses. Finally, it must be stressed that, despite the use of panel data, the paper estimates mainly associations, not the causal relationships between low SRH rates and recession-related economic variables.

## Conclusions

In this paper, we used balanced panel data from 2008 to 2011 EU-SILC database and regression-based decomposition methodology to understand how different economic mechanisms (changing income distribution, labour market developments, increases in material deprivation) have affected changes in the incidence of low SRH in Europe during the recent economic crisis. To our knowledge, this is the first study to estimate the separate impact of distinct recession-related economic factors on SRH in Europe. We have found that low SRH incidence increased during the GR by almost 2 percentage points for all EU countries, and by 3.8 percentage points for the Baltic countries. The recession-related economic factors together account for about 33% of the increase in low SRH incidence in Europe, and for about 100% of the increase in the Baltic countries. Our novel finding is that non-income poverty (material deprivation) has been an important factor worsening health during the recession as it accounted for 12% of the overall growth in low SRH in Europe (27% for of the Baltic countries). Future studies should investigate whether the link between increasing material deprivation and poorer SRH can also be found in case of other recessions.

## Additional file

Additional file 1: **Table A1.** Descriptive statistics for the sample of Baltic countries (Estonia, Lithuania, Latvia) (DOCX 42 kb)

## Abbreviations

EU: European Union
EU-SILC: European Union Statistics on Income and Living Conditions
GR: Great Recession
SRH: self-rated health
