# Supplementary material for: What accounts for the rise of low self-rated health during the recent economic crisis in Europe?
Source: Int J Equity Health. 2019 Jan 28;18:21. doi: 10.1186/s12939-019-0926-1 (PMC6350338; doi:10.1186/s12939-019-0926-1)
Supplement: Supplementary file 1 — Table A1. Descriptive statistics for the sample of Baltic countries (Estonia, Lithuania, Latvia) (DOCX 42 kb) [file 12939_2019_926_MOESM1_ESM.docx]

**Supplementary Appendix**

**Table A1.** Descriptive statistics for the sample of Baltic countries (Estonia, Lithuania, Latvia)

|  | 2008 | | 2011 | |
| --- | --- | --- | --- | --- |
|  | Mean | SD | Mean | SD |
| SRH status: 1-5 scale, 1=very bad, 5=very good | 3.474 | 0.806 | 3.389 | 0.800 |
| Very bad SRH | 0.016 | 0.124 | 0.018 | 0.133 |
| Very bad or bad SRH | 0.107 | 0.309 | 0.128 | 0.334 |
| Very bad, bad or fair SRH | 0.465 | 0.499 | 0.503 | 0.500 |
| Female | 0.604 | 0.489 | 0.595 | 0.491 |
| Age | 42.542 | 12.404 | 44.743 | 12.597 |
| Real equivalent household income (Euro in 2004 prices) | 9848.0 | 7625.9 | 8784.0 | 5558.6 |
| Poor (income < 60% of the median) | 0.208 | 0.406 | 0.189 | 0.392 |
| Rich (income > 200% of the median) | 0.105 | 0.307 | 0.105 | 0.306 |
| Relatively poor | 0.193 | 0.394 | 0.182 | 0.386 |
| Relatively rich | 0.084 | 0.278 | 0.074 | 0.262 |
| Material deprivation | 0.242 | 0.428 | 0.333 | 0.471 |
| Primary education and less | 0.010 | 0.100 | 0.011 | 0.102 |
| Lower secondary education | 0.118 | 0.323 | 0.120 | 0.325 |
| Upper secondary education | 0.588 | 0.492 | 0.574 | 0.495 |
| Tertiary education | 0.284 | 0.451 | 0.296 | 0.456 |
| Married | 0.605 | 0.489 | 0.567 | 0.496 |
| No longer married | 0.194 | 0.395 | 0.222 | 0.416 |
| Never married | 0.202 | 0.401 | 0.211 | 0.408 |
| Employed, full-time | 0.693 | 0.461 | 0.633 | 0.482 |
| Employed, part-time | 0.031 | 0.173 | 0.044 | 0.206 |
| Unemployed | 0.057 | 0.232 | 0.100 | 0.300 |
| Retired | 0.067 | 0.249 | 0.113 | 0.316 |
| Disabled | 0.051 | 0.221 | 0.064 | 0.246 |
| Inactive | 0.101 | 0.302 | 0.046 | 0.210 |
| Densely populated area | 0.436 | 0.496 | 0.463 | 0.499 |
| Intermediate populated area | 0.000 | 0.000 | 0.000 | 0.000 |
| Thinly populated area | 0.564 | 0.496 | 0.537 | 0.499 |
| *N* | 3719 | | | |

**Table A2.** Descriptive statistics for the sample of severe recession countries (Cyprus, Denmark, Estonia, Hungary, Iceland, Italy, Latvia, Lithuania, Portugal, Spain, and the UK)

|  | 2008 | | 2011 | |
| --- | --- | --- | --- | --- |
|  | Mean | SD | Mean | SD |
| SRH status: 1-5 scale, 1=very bad, 5=very good | 3.900 | 0.859 | 3.885 | 0.873 |
| Very bad SRH | 0.012 | 0.110 | 0.013 | 0.114 |
| Very bad or bad SRH | 0.062 | 0.241 | 0.070 | 0.256 |
| Very bad, bad or fair SRH | 0.263 | 0.440 | 0.265 | 0.441 |
| Female | 0.551 | 0.497 | 0.553 | 0.497 |
| Age | 44.096 | 11.988 | 46.209 | 12.194 |
| Real equivalent household income (Euro in 2004 prices) | 16549.5 | 11666.9 | 17452.8 | 13849.1 |
| Poor (income < 60% of the median) | 0.188 | 0.391 | 0.191 | 0.393 |
| Rich (income > 200% of the median) | 0.087 | 0.282 | 0.088 | 0.284 |
| Relatively poor | 0.136 | 0.343 | 0.131 | 0.338 |
| Relatively rich | 0.065 | 0.246 | 0.061 | 0.240 |
| Material deprivation | 0.153 | 0.360 | 0.167 | 0.373 |
| Primary education and less | 0.147 | 0.354 | 0.120 | 0.325 |
| Lower secondary education | 0.223 | 0.416 | 0.203 | 0.402 |
| Upper secondary education | 0.389 | 0.488 | 0.399 | 0.490 |
| Tertiary education | 0.241 | 0.428 | 0.279 | 0.448 |
| Married | 0.643 | 0.479 | 0.630 | 0.483 |
| No longer married | 0.119 | 0.324 | 0.131 | 0.337 |
| Never married | 0.238 | 0.426 | 0.239 | 0.427 |
| Employed, full-time | 0.564 | 0.496 | 0.506 | 0.500 |
| Employed, part-time | 0.099 | 0.298 | 0.108 | 0.311 |
| Unemployed | 0.057 | 0.232 | 0.088 | 0.283 |
| Retired | 0.083 | 0.277 | 0.124 | 0.329 |
| Disabled | 0.034 | 0.181 | 0.037 | 0.189 |
| Inactive | 0.163 | 0.369 | 0.136 | 0.343 |
| Densely populated area | 0.460 | 0.498 | 0.475 | 0.499 |
| Intermediate populated area | 0.281 | 0.449 | 0.291 | 0.454 |
| Thinly populated area | 0.260 | 0.439 | 0.234 | 0.424 |
| *N* | 18092 | | | |

**Table A3.** Logit regressions for low SRH, all countries

|  | 2008 | 2011 |
| --- | --- | --- |
| Female | -0.0365 | 0.0403 |
|  | (0.0582) | (0.0398) |
| Age 18-24 | -1.465^***^ | -1.591^***^ |
|  | (0.177) | (0.170) |
| Age 25-34 | -0.539^***^ | -0.879^***^ |
|  | (0.106) | (0.0820) |
| Age 45-54 | 0.598^***^ | 0.456^***^ |
|  | (0.0727) | (0.0481) |
| Age 55-64 | 0.803^***^ | 0.541^***^ |
|  | (0.0819) | (0.0472) |
| Real equivalent household income (Euro in 2004 prices) | -0.0171^***^ | -0.00720 |
|  | (0.00444) | (0.00625) |
| Poor (income < 60% of the median) | -0.00292 | 0.00826 |
|  | (0.0915) | (0.0803) |
| Rich (income > 200% of the median) | 0.153 | -0.158 |
|  | (0.135) | (0.138) |
| Relatively poor | 0.0120 | 0.0350 |
|  | (0.0924) | (0.0741) |
| Relatively rich | -0.00908 | 0.00236 |
|  | (0.132) | (0.0962) |
| Material deprivation | 0.711^***^ | 0.620^***^ |
|  | (0.0717) | (0.0524) |
| Primary education and less | 0.452^***^ | 0.790^***^ |
|  | (0.0852) | (0.0586) |
| Lower secondary education | 0.335^***^ | 0.369^***^ |
|  | (0.0718) | (0.0553) |
| Tertiary education | -0.291^***^ | -0.341^***^ |
|  | (0.0815) | (0.0547) |
| Married | -0.182^**^ | -0.239^***^ |
|  | (0.0795) | (0.0571) |
| No longer married | 0.0413 | -0.0600 |
|  | (0.0973) | (0.0691) |
| Employed, full-time | -0.288^***^ | -0.280^***^ |
|  | (0.0897) | (0.0670) |
| Unemployed | 0.309^**^ | 0.147 |
|  | (0.125) | (0.0905) |
| Retired | 0.385^***^ | 0.926^***^ |
|  | (0.110) | (0.0721) |
| Disabled | 1.519^***^ | 2.658^***^ |
|  | (0.144) | (0.134) |
| Inactive | 0.0445 | 0.213^**^ |
|  | (0.105) | (0.0830) |
| Missing information about labor market status | -0.200^***^ | 0.701 |
|  | (0.0689) | (0.651) |
| Thinly populated area | 0.0609 | -0.180^***^ |
|  | (0.0651) | (0.0428) |
| Densely populated area | 0.0153 | -0.0397 |
|  | (0.0622) | (0.0437) |
| Central-Western Europe | 0.0999 | 0.290^***^ |
|  | (0.0834) | (0.0871) |
| South-Eastern Europe | -0.399^***^ | -0.0649 |
|  | (0.0956) | (0.107) |
| Central-Eastern Europe | 0.573^***^ | 0.762^***^ |
|  | (0.0759) | (0.0790) |
| North-Western Europe | -0.0688 | -0.00879 |
|  | (0.0884) | (0.0824) |
| North-Eastern Europe | 1.075^***^ | 1.403^***^ |
|  | (0.0840) | (0.0981) |
| South-Western Europe | 0.00767 | -0.0824 |
|  | (0.0562) | (0.0570) |
| Constant | -1.121^***^ | -1.237^***^ |
|  | (0.157) | (0.154) |
| *N* | 43456 | |

*Note*: Standard errors in parentheses. ^*^ *p* < 0.1, ^**^ *p* < 0.05, ^***^ *p* < 0.01. Omitted categories are: age 35-44, upper secondary education, never married, part-time employed, intermediate populated area, Western Europe.

**Table A4.** Logit regressions for low SRH, Baltic countries

|  | 2008 | 2011 |
| --- | --- | --- |
| Female | 0.106 | 0.103 |
|  | (0.109) | (0.111) |
| Age 18-24 | -1.448^***^ | -1.577^***^ |
|  | (0.280) | (0.341) |
| Age 25-34 | -1.241^***^ | -0.868^***^ |
|  | (0.201) | (0.196) |
| Age 45-54 | 0.448^***^ | 0.887^***^ |
|  | (0.122) | (0.128) |
| Age 55-64 | 1.220^***^ | 1.367^***^ |
|  | (0.152) | (0.143) |
| Real equivalent household income (Euro in 2004 prices) | -0.0396^**^ | -0.0207 |
|  | (0.0168) | (0.0192) |
| Poor (income < 60% of the median) | -0.225 | -0.00260 |
|  | (0.196) | (0.237) |
| Rich (income > 200% of the median) | 0.606^*^ | -0.220 |
|  | (0.315) | (0.274) |
| Relatively poor | 0.369^*^ | -0.0979 |
|  | (0.202) | (0.233) |
| Relatively rich | -0.465^*^ | 0.377 |
|  | (0.249) | (0.281) |
| Material deprivation | 0.358^***^ | 0.660^***^ |
|  | (0.130) | (0.122) |
| Primary education and less | 1.049^*^ | 0.514 |
|  | (0.562) | (0.766) |
| Lower secondary education | 0.359^**^ | 0.171 |
|  | (0.179) | (0.198) |
| Tertiary education | -0.450^***^ | -0.689^***^ |
|  | (0.129) | (0.132) |
| Married | 0.373^**^ | 0.250 |
|  | (0.154) | (0.156) |
| No longer married | 0.239 | 0.220 |
|  | (0.175) | (0.180) |
| Employed, full-time | -0.352 | -0.511^**^ |
|  | (0.291) | (0.219) |
| Unemployed | 0.261 | -0.220 |
|  | (0.352) | (0.259) |
| Retired | 0.479 | 1.806^***^ |
|  | (0.394) | (0.289) |
| Disabled | 2.977^***^ | 3.889^***^ |
|  | (0.691) | (0.618) |
| Inactive | -0.297 | -0.166 |
|  | (0.330) | (0.325) |
| Thinly populated area | 0.0597 | -0.201^*^ |
|  | (0.104) | (0.106) |
| Densely populated area | - | - |
|  |  |  |
| Latvia | 0.586^***^ | 0.432^***^ |
|  | (0.110) | (0.131) |
| Lithuania | 0.247^**^ | 0.260^**^ |
|  | (0.117) | (0.130) |
| Constant | -0.375 | -0.379 |
|  | (0.352) | (0.339) |
| *N* | 3719 | 3719 |

*Note*: Standard errors in parentheses. ^*^ *p* < 0.1, ^**^ *p* < 0.05, ^***^ *p* < 0.01. Omitted categories are: age 35-44, upper secondary education, never married, part-time employed, intermediate populated area, Estonia.

**Table A5.** Logit regressions for low SRH, severe recession countries

|  | 2008 | 2011 |
| --- | --- | --- |
| Female | 0.187^*^ | 0.154 |
|  | (0.105) | (0.0997) |
| Age 18-24 | -1.429^***^ | -2.020^***^ |
|  | (0.296) | (0.431) |
| Age 25-34 | -0.662^***^ | -0.995^***^ |
|  | (0.175) | (0.202) |
| Age 45-54 | 0.462^***^ | 0.297^**^ |
|  | (0.119) | (0.117) |
| Age 55-64 | 0.778^***^ | 0.510^***^ |
|  | (0.139) | (0.119) |
| Real equivalent household income (Euro in 2004 prices) | -0.0451^***^ | -0.0137 |
|  | (0.0148) | (0.0114) |
| Poor (income < 60% of the median) | -0.207 | 0.127 |
|  | (0.178) | (0.174) |
| Rich (income > 200% of the median) | 0.844^***^ | -0.0420 |
|  | (0.257) | (0.235) |
| Relatively poor | -0.184 | 0.0630 |
|  | (0.166) | (0.170) |
| Relatively rich | 0.0370 | 0.250 |
|  | (0.207) | (0.193) |
| Material deprivation | 0.680^***^ | 0.632^***^ |
|  | (0.126) | (0.126) |
| Primary education and less | 0.203 | 0.445^***^ |
|  | (0.144) | (0.145) |
| Lower secondary education | -0.192 | 0.182 |
|  | (0.145) | (0.148) |
| Tertiary education | -0.508^***^ | -0.432^***^ |
|  | (0.142) | (0.129) |
| Married | 0.0768 | -0.122 |
|  | (0.145) | (0.146) |
| No longer married | 0.0913 | 0.0480 |
|  | (0.186) | (0.181) |
| Employed, full-time | -0.596^***^ | -0.464^**^ |
|  | (0.228) | (0.212) |
| Unemployed | -0.0261 | -0.0753 |
|  | (0.257) | (0.238) |
| Retired | 0.322 | 1.053^***^ |
|  | (0.272) | (0.230) |
| Disabled | 3.024^***^ | 3.185^***^ |
|  | (0.468) | (0.402) |
| Inactive | -0.193 | 0.322 |
|  | (0.230) | (0.229) |
| Thinly populated area | 0.0697 | -0.00685 |
|  | (0.156) | (0.157) |
| Densely populated area | -0.0870 | 0.166 |
|  | (0.153) | (0.155) |
| Spain | 0.665^***^ | 0.464^***^ |
|  | (0.112) | (0.115) |
| Estonia | 1.450^***^ | 1.918^***^ |
|  | (0.150) | (0.143) |
| Lithuania | 1.700^***^ | 2.204^***^ |
|  | (0.152) | (0.143) |
| Latvia | 1.992^***^ | 2.271^***^ |
|  | (0.136) | (0.140) |
| Constant | -1.343^***^ | -2.086^***^ |
|  | (0.413) | (0.350) |
| *N* | 8206 | 8206 |

*Note*: Standard errors in parentheses. ^*^ *p* < 0.1, ^**^ *p* < 0.05, ^***^ *p* < 0.01. Omitted categories are: age 35-44, upper secondary education, never married, part-time employed, intermediate populated area, Greece.
